# *Vagococcus changpingensis* sp. nov., a Fly-Associated Bacterium with Human Gut Metagenomic Representatives: Genomic and Metagenomic Insights into Its Ecological Distribution

**DOI:** 10.3390/microorganisms14081748

**Published:** 2026-08-08

**Authors:** Ji Pu, Peng Cheng, Yumei Guo, Di Xiao, Huifang Zhang, Dong Jin

**Affiliations:** 1 National Institute for Communicable Disease Control and Prevention, Chinese Center for Disease Control and Prevention & Chinese Academy of Preventive Medicine, Changping District, Beijing 102206, China; puji@icdc.cn (J.P.); xiaodi@icdc.cn (D.X.); zhanghuifang@icdc.cn (H.Z.); 2 Huangshan Municipal Center for Disease Control and Prevention (Huangshan Municipal Health Supervision Institute), Huangshan 245000, China; hscdccp@163.com; 3 Hebei Key Laboratory of Intractable Pathogens, Shijiazhuang Center for Disease Control and Prevention, Shijiazhuang 050011, China; guoymcdc@163.com

**Keywords:** *Vagococcus changpingensis*, polyphasic taxonomy, metagenomic mining, pangenome analysis, human gut metagenome, cross-host adaptation

## Abstract

The genus *Vagococcus* comprises Gram-positive bacteria with a broad ecological distribution, yet its diversity and potential links between animal and human habitats remain underexplored. Here, we report two novel fly-associated strains, CY52-2^T^ and CY62-2, isolated from a retail market in Beijing, China. Polyphasic taxonomic analyses demonstrated that they represent a novel species, for which we propose the name *Vagococcus changpingensis* sp. nov. Large-scale mining of 805 public metagenomes identified two human gut-derived genomes that share > 99.3% ANI with *V. changpingensis*, extending the known distribution of this species from insects to the human gastrointestinal tract at the genomic level. Pangenome analysis revealed an open pangenome and uncovered niche-specific gene sets. These findings highlight the power of targeted metagenomics to reveal the potential ecological breadth of newly described species and provide a genomic framework for future investigations of the genus *Vagococcus*.

## 1. Introduction

The genus *Vagococcus* (family *Vagococcaceae*) comprises Gram-positive, catalase-negative, facultatively anaerobic cocci that inhabit a remarkably diverse range of ecological niches [1,2]. Currently, 23 species with validly published names are recognized within this genus (LPSN, accessed January 2026) [3]. Type strains of this genus have been recovered from an array of sources, including animal-derived foods such as ground beef (*V. bubulae*, *V. carniphilus*), fermented seafood (*V. jeotgali*), and sour milk (*V. teuberi*), as well as from insects and their habitats, including beetle larvae (*V. allomyrinae*), wasp digestive tracts (*V. entomophilus*), and blow flies (*V. luciliae*). This broad ecological versatility, spanning food matrices and insect hosts, hints at potential transmission routes through food chains or insect vectors that remain largely unexplored.

The clinical relevance of *Vagococcus* is increasingly recognized [4]. Although infections are relatively uncommon, the type species *V. fluvialis* has been implicated in serious conditions such as infective endocarditis [5,6], and other species have been linked to wound infections and animal diseases. This accumulating evidence underscores the potential role of *Vagococcus* species as opportunistic pathogens and raises the question of whether animals, including insects, may serve as reservoirs for human-associated *Vagococcus* lineages. Insects, particularly synanthropic flies, are well-established mechanical vectors of pathogenic and commensal bacteria, capable of bridging contaminated environments and human habitats. Flies sampled from retail food markets represent a sentinel interface where environmental, foodborne, and human-associated microorganisms converge. Despite the broad distribution of *Vagococcus*, the full extent of its diversity and its potential presence in human-associated environments remain underexplored.

In this study, we isolated two novel *Vagococcus* strains, CY52-2^T^ and CY62-2, from flies collected at a retail market in Beijing, China. Using a polyphasic taxonomic approach integrating phylogenomic, phenotypic, and chemotaxonomic evidence, we demonstrate that these strains represent a novel species, for which the name *Vagococcus changpingensis* sp. nov. is proposed. Given the isolation of related *Vagococcus* species from animal sources and the increasing recognition of environmental bacteria in the human microbiome, we hypothesized that *Vagococcus* species may also be present in the human gastrointestinal tract. By combining large-scale metagenomic screening with pangenome analysis, we further aimed to uncover the broader ecological distribution of this novel species and identify genomic signatures potentially associated with different host environments.

## 2. Materials and Methods

### 2.1. Sample Collection and Strain Isolation

On 20 August 2016, insect samples (including flies and cockroaches), raw pork, and environmental swabs were collected from a retail raw pork market in Beijing, China, as part of a broader investigation [7]. Samples were transported at 4 °C and processed within 24 h. To increase detection sensitivity, flies were divided into groups of approximately 20 individuals each. The pooled flies in each group were mechanically homogenized, yielding 20 pooled homogenate samples (designated CY51–CY70). The primary isolation was performed as part of a broader surveillance study targeting *Listeria monocytogenes* and *Enterococcus* spp. in retail meat and environmental samples. Each homogenized fly pool was enriched in 225 mL of Half Fraser broth (Oxoid Ltd., Hampshire, UK) at 30 °C for 24 h with shaking at 220 rpm. A 1 mL aliquot of the primary culture was then transferred to 9 mL of Fraser broth (Oxoid Ltd.) and incubated at 37 °C for 48 h under the same shaking conditions. Enriched cultures were streaked onto KF Streptococcus agar (Beijing Land Bridge Technology, Beijing, China) and incubated aerobically at 37 °C with 5% CO_2_ for 48 h to isolate *Enterococcus* for antimicrobial resistance surveillance. During this screening, two colonies exhibiting morphology distinct from typical enterococci were observed on KF Streptococcus agar plates from two independent fly pools (CY52 and CY62). These colonies were selected for further purification by restreaking onto Columbia agar supplemented with 5% defibrinated sheep blood (bioMérieux, Marcy-l’Étoile, France) and incubated under the same conditions for 24 h. Pure cultures were stored at −80 °C in brain heart infusion broth (Oxoid Ltd.) supplemented with 20% (*v*/*v*) glycerol. Since this study involved only environmental samples and no human subjects or animal experiments, no ethical approval was required.

### 2.2. 16S rRNA Gene Sequencing and Preliminary Identification

Genomic DNA was extracted from purified colonies using Lysis Buffer for Microorganism (TaKaRa, Dalian, China) according to the manufacturer’s instructions. The nearly full-length 16S rRNA gene was amplified by PCR using universal bacterial primers [8]. Amplicons were sequenced by Sanger sequencing at Beijing Tianyihuiyuan Biotechnology Co., Ltd. (Beijing, China) and assembled using DNASTAR version 7 (DNASTAR, Madison, WI, USA). For preliminary taxonomic assignment, the obtained sequences were compared against the EzBioCloud database (https://www.ezbiocloud.net/; accessed on 10 December 2025) [9]. Strains meeting either of the following criteria were selected for downstream polyphasic analysis: (i) sequence similarity < 98.7% to the closest validly published type strain; (ii) sequence similarity ≥ 98.7% but equally matching two or more different species.

### 2.3. Genome Sequencing, Assembly, and Annotation

Genomic DNA for whole-genome sequencing was extracted using the Wizard Genomic DNA Purification Kit (Promega Corporation, Madison, WI, USA). For strains CY52-2^T^ and CY62-2, Illumina libraries were prepared and sequenced on a MiSeq platform (Illumina, San Diego, CA, USA), generating 2 × 250 bp paired-end reads. Raw reads were quality-filtered and adapter-trimmed using Trimmomatic v0.33. For strain CY62-2, a total of 1179 Mb of raw data were generated. After quality filtering, 1000 Mb of clean data were retained, representing 84.8% of the raw data. The clean data exhibited a GC content of 33.9% and Q20 and Q30 values of 97.73% and 92.94%, respectively, indicating high sequencing quality. The high-quality reads were then de novo assembled into contigs using SPAdes v3.6.2, and assembly gaps were subsequently closed to improve continuity. Strain CY52-2^T^ was selected as the type strain because it was the first isolate recovered from the fly samples, and to obtain a complete, closed reference genome, it was additionally sequenced using single-molecule real-time (SMRT) technology on the PacBio RS II platform (Pacific Biosciences, Menlo Park, CA, USA). Long reads were assembled using SMRT Analysis v2.3.0, yielding a single circular chromosome of 2,019,321 bp (G + C content, 33.7%) and one circular plasmid of 78,932 bp, without gaps. Circularization of both replicons was confirmed by the presence of overlapping terminal sequences in the assembled contigs, as identified by the SMRT Analysis pipeline. The mean sequencing coverage was 210×, and only 0.02% of bases were missing, resulting in an N50 of 2,019,350 bp. Genome annotation for all genomes used in this study was performed using Prokka v1.14.6 [10] with default parameters; for strain CY52-2^T^, this predicted 1893 genes, with a coding density of 87.5%.

### 2.4. Genome-Based Taxonomic Delineation

Species boundaries were assessed using two complementary whole-genome metrics. Digital DNA–DNA hybridization (dDDH) values were calculated using the Type (Strain) Genome Server (TYGS; https://tygs.dsmz.de) [11]. The TYGS pipeline (accessed on 26 December 2025) identified the closest type strain genomes via MASH and 16S rDNA BLAST approaches and refined intergenomic distances using the Genome BLAST Distance Phylogeny (GBDP) method with default parameters. Pairwise average nucleotide identity (ANI) values were calculated using FastANI v1.33 (accessed 26 December 2025) against all available *Vagococcus* type strain genomes. In addition to the TYGS analysis, in silico DDH values were independently estimated using the Genome-to-Genome Distance Calculator (GGDC) v3.0 (accessed 26 December 2025) with the recommended formula d_4_—which shows the best correlation with wet-lab DDH and provides robust confidence intervals—to include all validly published type strains of the genus *Vagococcus* and provide complementary validation.

### 2.5. Phylogenomic and Pangenome Analyses

The 16S rRNA gene sequences of strains CY52-2^T^ and CY62-2 were aligned with those of closely related type strains using SINA, filtered using trimAl and a maximum-likelihood (ML) phylogenetic tree was reconstructed with IQ-TREE using 1000 bootstrap replicates. The final trimAl-filtered alignment contained 1557 nucleotide sites. The tree was reconstructed in IQ-TREE using the TVM + F + I + G4 model selected by ModelFinder according to BIC. For phylogenomic inference, the genomes of strains CY52-2^T^, CY62-2, and all 23 validly published *Vagococcus* type strains were annotated with Prokka (v1.14.6). Core genes, defined as those present in ≥95% of genomes, were identified using Roary v3.13.0 [12] with a 75% amino acid identity threshold. Core gene sequences were aligned with MAFFT (v7.505) [13], and an ML phylogenomic tree was constructed using IQ-TREE (v2.2.2) [14] with automatic selected best-fit model GTR + F + I + R5 according to BIC and 1000 ultrafast bootstrap replicates. Pangenome analysis was performed using the same Roary output. The gene presence/absence matrix was imported into R for downstream analysis. Pangenome accumulation curves and heatmaps were visualized using custom R scripts (v4.2.3) employing the ggplot2 package (v3.4.2).

### 2.6. Targeted Metagenomic Mining for Ecological Delineation

To investigate the broader ecological distribution of the novel species, a targeted metagenomic mining pipeline was deployed. First, the GTDB was directly queried for genomes with high ANI values to strain CY52-2^T^. Concurrently, metagenomic datasets from fly hosts and human gut environments were retrieved from the NCBI Sequence Read Archive (SRA). Fly-associated metagenomes were identified using the query ((fly OR “*Musca domestica*” OR “*Hermetia illucens*” OR “*Drosophila*”) AND “metagenome” [All Fields]) NOT “amplicon” [All Fields] AND “strategy wgs” [Properties]. Human gut metagenomic datasets were retrieved using the following search query—(“human gut metagenome”[Organism] OR “human feces metagenome”[Organism]) AND “Beijing”[All Fields] NOT “amplicon”[All Fields] AND “strategy wgs”[Properties]—with the search restricted to samples from Beijing due to their geographical relevance to our isolated strains. Raw sequencing data were downloaded via the European Nucleotide Archive (ENA) FTP server using Kingfisher (0.5.0) [15].

To avoid computationally prohibitive whole-community binning, a rapid two-pronged screening strategy was applied. First, 16S rRNA gene fragments were extracted from raw metagenomic reads using phyloFlash v3.4 [16] against the SILVA database (release 138.1) and aligned against known *Vagococcus* references (genus-level threshold: 94.5% similarity). Paired-end raw reads were mapped against the complete genome of strain CY52-2^T^ using Bowtie2 v2.5.4 with the very-sensitive preset in the default end-to-end mode (-D 20 -R 3 -N 0 -L 20 -i S,1,0.50). No fixed percentage identity, minimum aligned-length, or mapping-quality threshold was specified. Valid alignments were accepted according to the default Bowtie2 end-to-end minimum-score function (-score-min L,-0.6,-0.6), with the entire read participating in the alignment. Unmapped and secondary alignment records were removed using SAMtools v1.21 (view -F 4 -F 256). The retained reads were name-sorted, converted to paired FASTQ files using BEDTools v2.31.1 and assembled using SPAdes v4.0.0 with the --meta and --phred-offset 33 options. These screening approaches were used to prioritize datasets for binning, whereas final taxonomic assignments were determined using genome-wide ANI and core-gene phylogenetic analyses.

For the selected metagenomes, raw reads were quality-controlled using fastp [17]. Low-quality reads were removed based on the following criteria: length < 100 bp, >5 ambiguous bases (N), and average Phred quality score < 20. Sliding window quality trimming (window size = 4, mean quality threshold = 20) was applied to both the 5′ and 3′ ends of the reads. To prevent misassembly caused by host DNA contamination, the high-quality reads were mapped against the host reference database using Bowtie2. This reference database comprised the genomes of *Musca domestica*, *Hermetia illucens*, *Drosophila melanogaster* and *Homo sapiens*, which were retrieved via NCBI Datasets. Strictly unmapped read pairs were extracted using SAMtools (1.23.1) (parameters -f 12 -F 256) and converted back to FASTQ format using BEDTools. The host-depleted, high-quality metagenomic reads were de novo assembled into contigs using metaSPAdes (v4.3.0) with the --meta flag. Finally, MAGs (metagenome-assembled genomes) were reconstructed from the assembled scaffolds using BASALT [18]. The binning module was executed under the “sensitive” mode with “deep” refinement parameters. During the initial exploratory recovery, MAGs with ≥50% completeness and ≤10% contamination were retained to avoid prematurely excluding potentially informative *Vagococcus* candidates. Following provisional genus-level classification, only MAGs with >90% completeness and <10% contamination were included in downstream ANI-based taxonomic assignment, core-gene phylogenetic analysis, and pangenome analysis, because sufficiently complete genomes were required for reliable genome-wide comparisons. MAGs that did not meet these stricter criteria were excluded from downstream genome-scale analyses.

### 2.7. MALDI-TOF MS Analysis

Strains CY52-2^T^ and CY62-2 were analyzed by matrix-assisted laser desorption/ionization time-of-flight mass spectrometry (MALDI-TOF MS) using a Microflex LRF system (Bruker Daltonics, Bremen, Germany). Spectra were acquired in linear mode with an N_2_ laser, accumulating 500 shots per sample. The instrument was calibrated using the China CDC standard calibration kit [19] prior to each run. This kit was developed in-house, has been patented, and is routinely shared among public health laboratories within the China CDC system; it is not commercially available but can be obtained upon reasonable request for collaborative or research purposes. Data were analyzed with FlexControl v3.4 and MALDI Biotyper Compass Explorer v4.1 (Bruker Daltonics). Main Spectrum Profile (MSP) dendrograms were constructed to visualize clustering relationships among 15 *Vagococcus* reference strains (13 *V. fluvialis* and 2 *V. lutrae* strains) present in the Biotyper database.

### 2.8. Phenotypic and Biochemical Characterization

Colony morphology was observed after 24 h of aerobic incubation at 37 °C on Columbia agar with 5% defibrinated sheep blood. Cell morphology and Gram reaction were determined by light microscopy (Gram stain) and transmission electron microscopy (TEM). Catalase activity was tested using the ID Colour Catalase kit (bioMérieux, Marcy-l’Étoile, France). Motility was assessed on soft agar plates (0.4% agar). Growth at 10, 22, 35, 37, and 45 °C was evaluated in brain heart infusion broth (Oxoid Ltd.) with pH adjusted to 7.5 [20], and scored as positive (OD600 ≥ 0.3), weak (OD600 0.1–0.3), or negative (OD600 < 0.1) after 48 h of incubation. Salt tolerance was tested in the same medium supplemented with 6.5% (w/v) NaCl [20]. Biochemical profiles were determined using the API 20 Strep and API 50 CH systems (bioMérieux) following the manufacturer’s protocols, with the latter using API 50 CHL suspension medium. The API 20 Strep and API 50 CH strips were incubated at 37 °C for 24 h and 48 h, respectively. Results were interpreted according to the manufacturer’s colorimetric chart after 24 h (API 20 Strep) and 48 h (API 50 CH). All phenotypic tests were performed in duplicate.

## 3. Results

### 3.1. Strain Isolation and Preliminary Identification

After 48 h of aerobic incubation with 5% CO_2_ at 37 °C on KF Streptococcus agar, colonies of strain CY52-2^T^ are approximately 1.0 mm in diameter, circular, greyish-white, with irregular margins and a rough, dry surface. On Columbia agar supplemented with 5% defibrinated sheep blood, colonies are similar in size and shape but appear smooth, with entire margins and α-hemolytic activity after 24 h of incubation.

The nearly full-length 16S rRNA gene sequences (1480 nt) were obtained for strains CY52-2^T^ and CY62-2, which were found to be identical. Comparative analysis against the type strains of closely related species in the EzBioCloud database revealed the highest sequence similarity to *Vagococcus luciliae* strain G314F^T^ (99.6%) and *V. bubulae* SS1994^T^ (99.3%). The similarity to other phylogenetically neighbouring type strains ranged from 98.1% to 98.6%, including *V. martis* D7T301^T^ (98.6%), *V. teuberi* DSM 21459^T^ (98.6%), *V. silagei* 2B-2^T^ (98.4%), *V. vulneris* SS1995^T^ (98.4%), and *V. penaei* CD276^T^ (98.1%).

The draft genome sequences of strains CY52-2^T^ and CY62-2 were submitted to the Type (Strain) Genome Server (TYGS) for genome-based taxonomic analysis. Digital DNA–DNA hybridization (dDDH) values were calculated using the recommended GGDC formula d_4_. The dDDH value between CY52-2^T^ and CY62-2 was 94.8% (confidence interval: 93.1–96.0%), confirming that they represent the same species. In contrast, the dDDH value between the proposed type strain CY52-2^T^ and its closest phylogenetic relative, *V. luciliae* G314F^T^, was 52.2% (49.5–54.8%), followed by 36.6% (34.1–39.1%) with *V. bubulae* SS1994^T^. These values are well below the 70% threshold widely accepted for prokaryotic species delineation. The dDDH values between CY52-2^T^ and all other recognized type strains of the genus *Vagococcus* and closely related *Enterococcus* species were even lower, ranging from 19.0% to 34.0% (Supplementary Table S1).

### 3.2. Genome-Based Species Delineation

Strain CY52-2 was selected as the proposed type strain of the novel species and was subjected to complete genome sequencing using the PacBio platform to obtain a closed circular genome. For comparative genomic analyses, the draft genome of strain CY62-2 was also included. Pairwise average nucleotide identity (ANI) values were calculated between strains CY52-2^T^, CY62-2, and the type strains of all recognized *Vagococcus* species. The ANI value between CY52-2^T^ and CY62-2 was 99.4%, confirming that they represent the same species. Compared with CY52-2^T^, the highest ANI value among the *Vagococcus* type strains was 94.1% with *V. luciliae* G314F^T^ (GCA024637875). Three species, namely *V. teuberi* (CP017267), *V. bubulae* SS1994^T^ (GCA003950315), and *V. martis* D7T301^T^ (MVAB01000001) showed ANI values 89.53%, 89.62%, and 88.64% relative to CY52-2^T^, respectively (Table 1). Digital DNA–DNA hybridization (dDDH) values were also calculated between strains CY52-2^T^, CY62-2, and all other *Vagococcus* type strains. The results were consistent with the ANI-based findings and the TYGS analysis. Apart from *V. luciliae* G314F^T^, all other *Vagococcus* species showed substantially lower dDDH values with CY52-2^T^, ranging from 18.6% to 36.8% (Supplementary Table S2), all well below the 70% threshold for prokaryotic species delineation.

### 3.3. Metagenomic Recovery Expands the Ecological Range

To explore whether the novel species might inhabit environments beyond the fly samples, a targeted metagenomic mining strategy was employed. The two human gut-derived genomes, MGYG-HGUT-02299 and OM08-11BH, share >99.3% ANI with strain CY52-2^T^ and were originally deposited under provisional labels (Table 1). Initial queries against the GTDB identified two genomes that shared > 99.3% ANI with strain CY52-2^T^, far exceeding the 95% species threshold. These genomes, originally deposited as *Enterococcus bacterium* and *Melissococcus* sp., respectively, were thus conclusively identified as belonging to the novel taxon and were reassigned to *V. changpingensis*.

**Figure 1:**
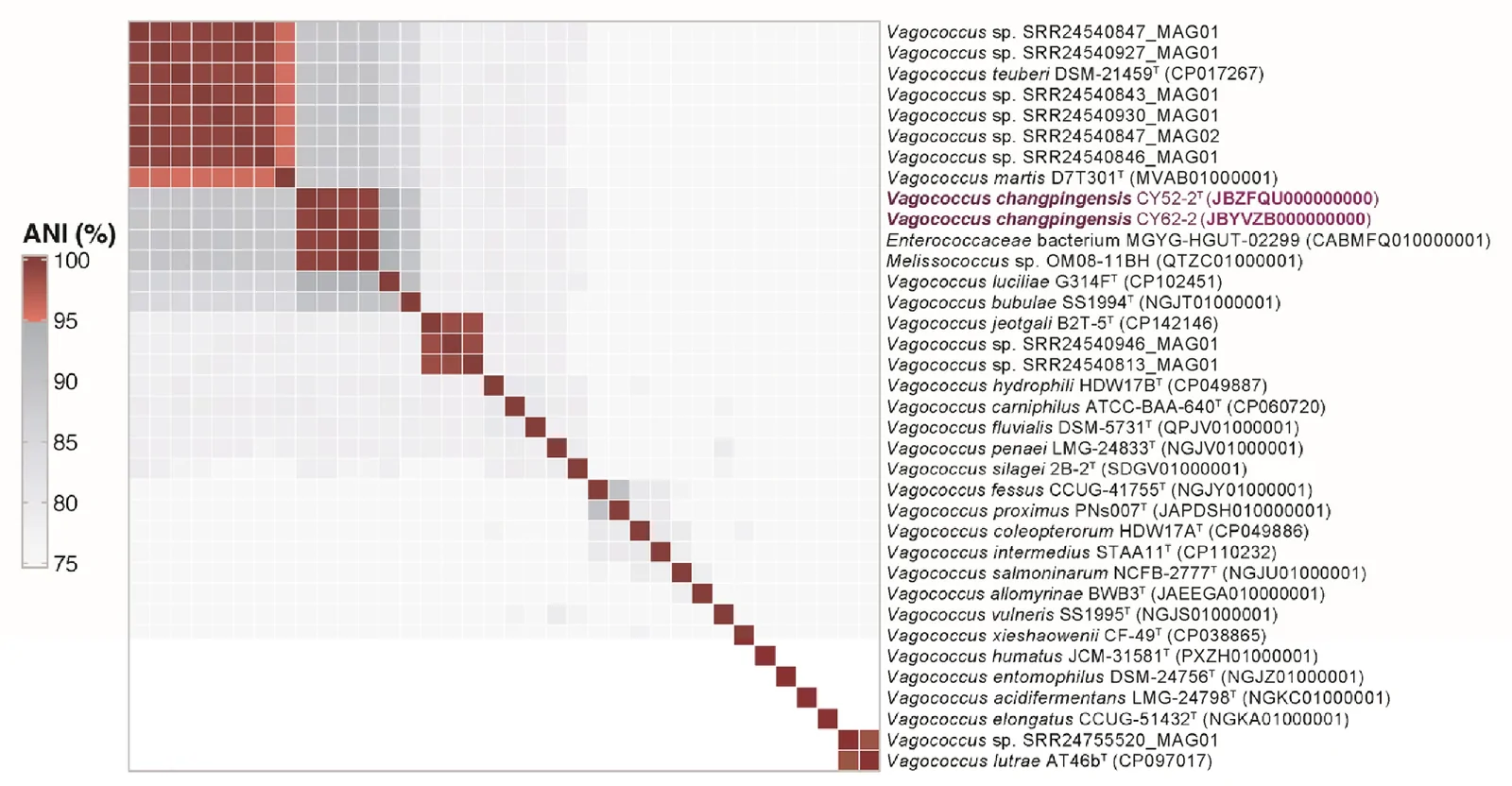
Average nucleotide identity among *Vagococcus changpingensis* and related *Vagococcus* genomes. Pairwise average nucleotide identity (ANI) values were displayed as a lower-triangular heatmap. The colour scale ranges from 75% to 100%, with darker red indicating higher ANI values. The 95% value represents the commonly accepted threshold for prokaryotic species delineation. Strains *V. changpingensis* CY52-2^T^ and CY62-2 are highlighted in purple. The human gut-derived genomes MGYG-HGUT-02299 and OM08-11BH cluster with the two isolates and exhibit ANI values above the species-level threshold.

In parallel, 376 fly-associated and 429 human gut-associated metagenomic datasets were screened using a two-pronged approach combining 16S rRNA gene profiling and targeted read mapping against the CY52-2^T^ reference genome. A total of 31 fly-associated metagenomes yielded positive signals and were subjected to de novo assembly and binning (Supplementary Table S3). This yielded 1634 MAGs, of which 49 were classified as *Vagococcus* spp. Nine MAGs fulfilled the downstream analysis criteria of >90% completeness and <10% contamination and were retained for ANI-based taxonomic assignment, core-genome phylogenetic analysis, and pangenome analysis. The remaining 40 candidates were excluded from genome-scale analyses because of insufficient completeness and/or excessive contamination and therefore did not contribute to the taxonomic or ecological conclusions (Supplementary Tables S4 and S5). Strikingly, ANI comparisons revealed that none of these nine fly-derived MAGs belonged to *V. changpingensis*. Instead, six exhibited >99% ANI with *V. teuberi* DSM 21459^T^, two clustered with *V. jeotgali* B2T-5^T^, and one (SRR24755520_MAG01) clustered with *V. lutrae* AT46b (Figure 1). These results indicate that diverse *Vagococcus* lineages coexist within the fly microbiome, but *V. changpingensis* was not detected in the fly metagenomes from these public datasets.

### 3.4. Phylogenetic and Phylogenomic Placement

**Figure 2:**
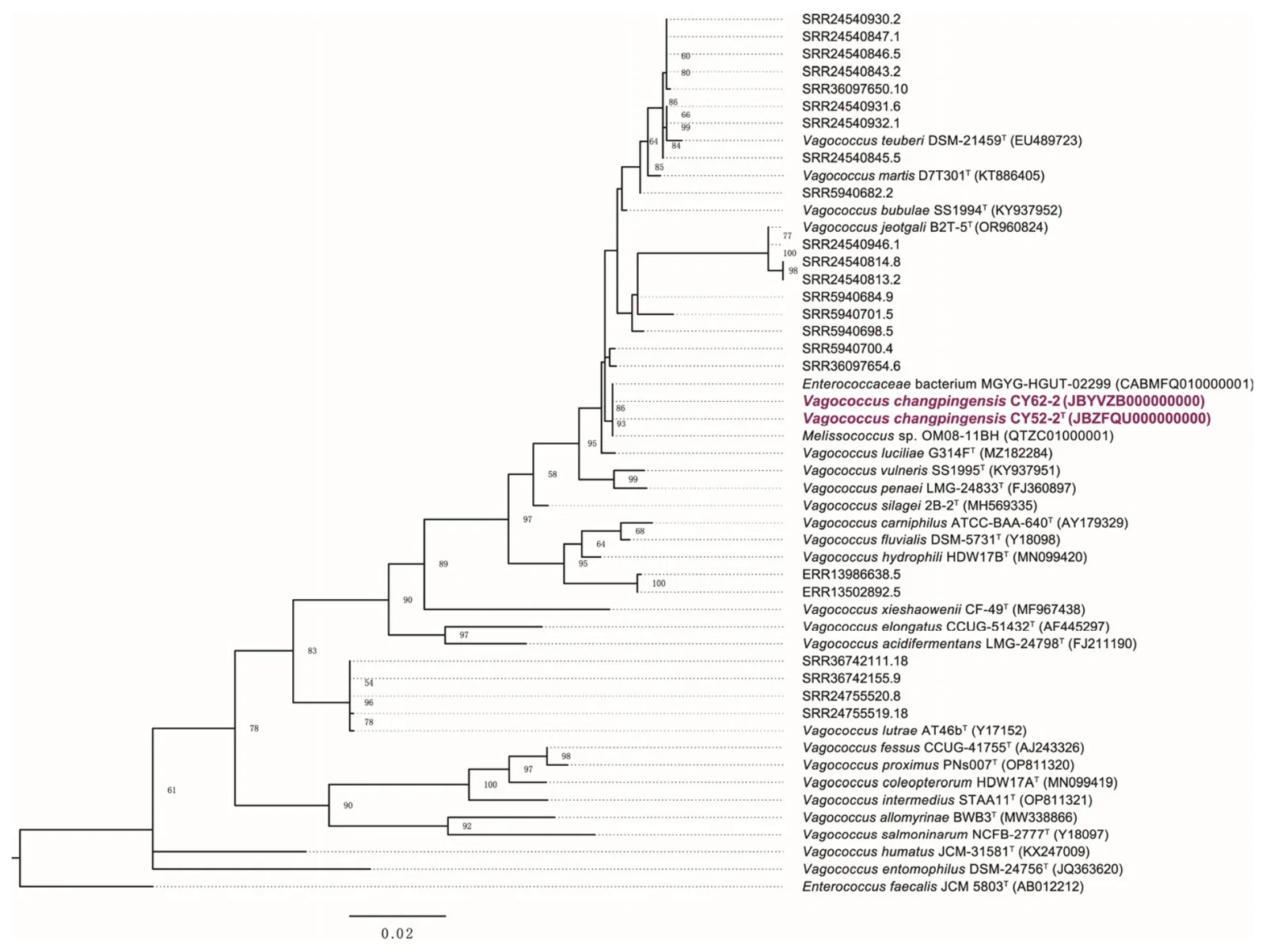
Maximum-likelihood phylogenetic tree based on 16S rRNA gene sequences showing the position of strains CY52-2^T^ and CY62-2 within the genus *Vagococcus*. Bootstrap values (≥50%) based on 1000 replicates are indicated at nodes. Bar, 0.01 substitutions per nucleotide position. The 16S rRNA gene sequence of *Enterococcus faecalis* JCM 5803^T^ (accession number: AB012212) was used as an outgroup.

The phylogenetic position of the novel species was assessed using both 16S rRNA gene and core genome sequences, incorporating the two human gut-derived MAGs and the nine newly recovered fly-associated *Vagococcus* MAGs alongside the type strains. Maximum-likelihood analysis of 16S rRNA gene sequences placed strains CY52-2^T^, CY62-2, and the two human gut MAGs in a tightly clustered, well-supported subclade within the genus *Vagococcus*, sister to the clade comprising *V. luciliae* G314F^T^ and *V. bubulae* SS1994^T^ (Figure 2).

**Figure 3:**
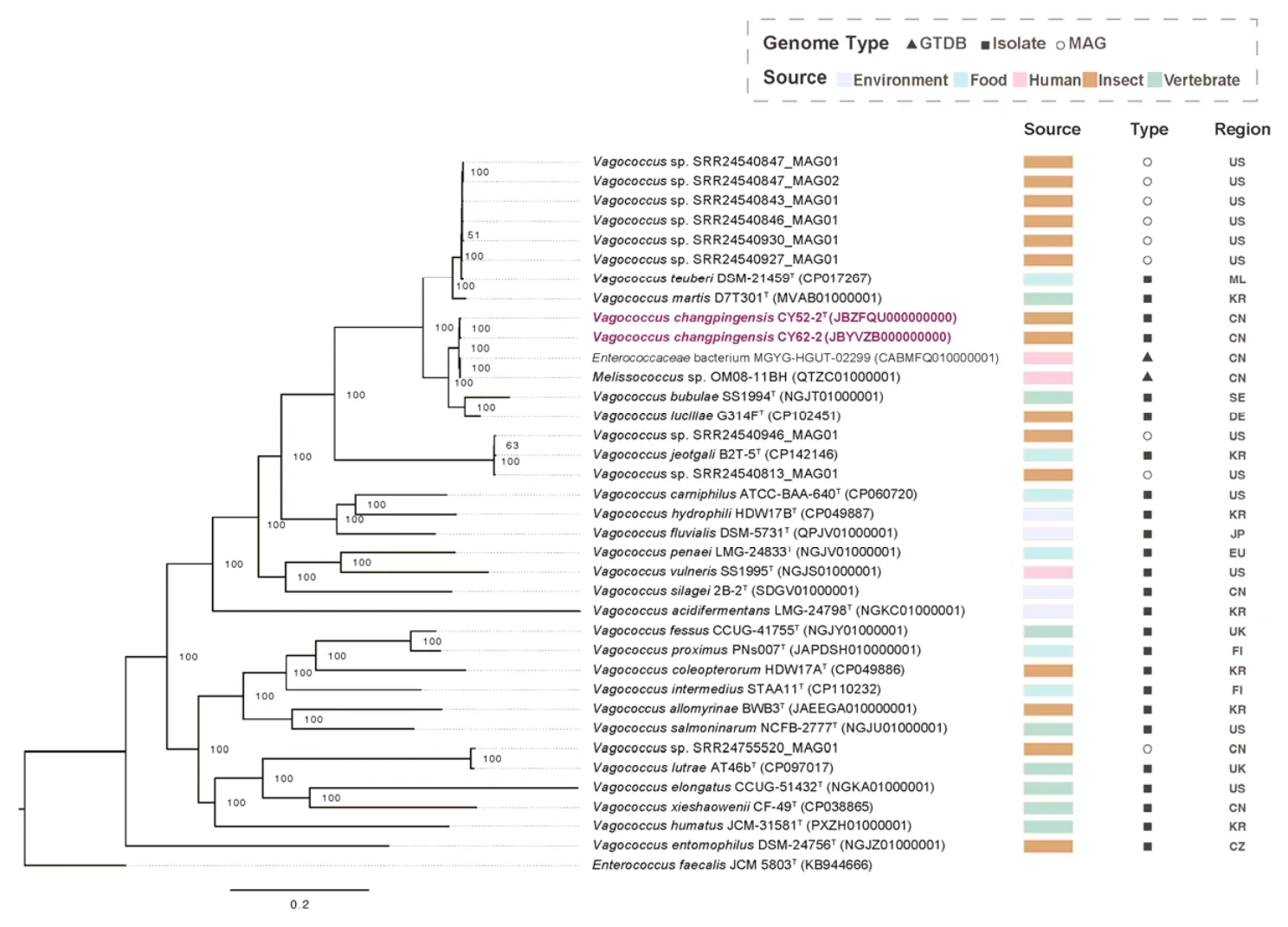
Phylogenomic relationships among *Vagococcus changpingensis* and related *Vagococcus* genomes. The maximum-likelihood tree was reconstructed in IQ-TREE from concatenated core gene sequences aligned using MAFFT, with automatic best-fit model selection and 1000 ultrafast bootstrap replicates. Bootstrap support values are shown at the nodes, and the scale bar represents 0.2 substitutions per site. *Enterococcus faecalis* JCM 5803^T^ was used as the outgroup. Symbols indicate genome type (triangle, GTDB genome; square, isolate; circle, MAG), while colours indicate isolation-source categories. Region abbreviations: CN, China; CZ, Czech Republic; DE, Germany; EU, Europe; FI, Finland; JP, Japan; KR, Republic of Korea; ML, Mali; SE, Sweden; UK, United Kingdom; and US, United States. Strains CY52-2^T^ and CY62-2 are highlighted in purple.

The 16S rRNA sequences from the fly-associated MAGs clustered accurately with their respective reference species (*V. teuberi* and *V. jeotgali*), confirming the reliability of the screening pipeline. Phylogenomic analysis based on 217 concatenated core genes contained 216,928 nucleotide positions further reinforced this topology. The maximum-likelihood core genome tree placed strains CY52-2^T^, CY62-2, and the two human gut-derived genomes in a robust, distinct monophyletic clade (Figure 3), strongly supporting their classification as a single species distinct from all currently recognized *Vagococcus* taxa.

### 3.5. Pangenome Architecture and Niche-Specific Gene Signatures

**Figure 4:**
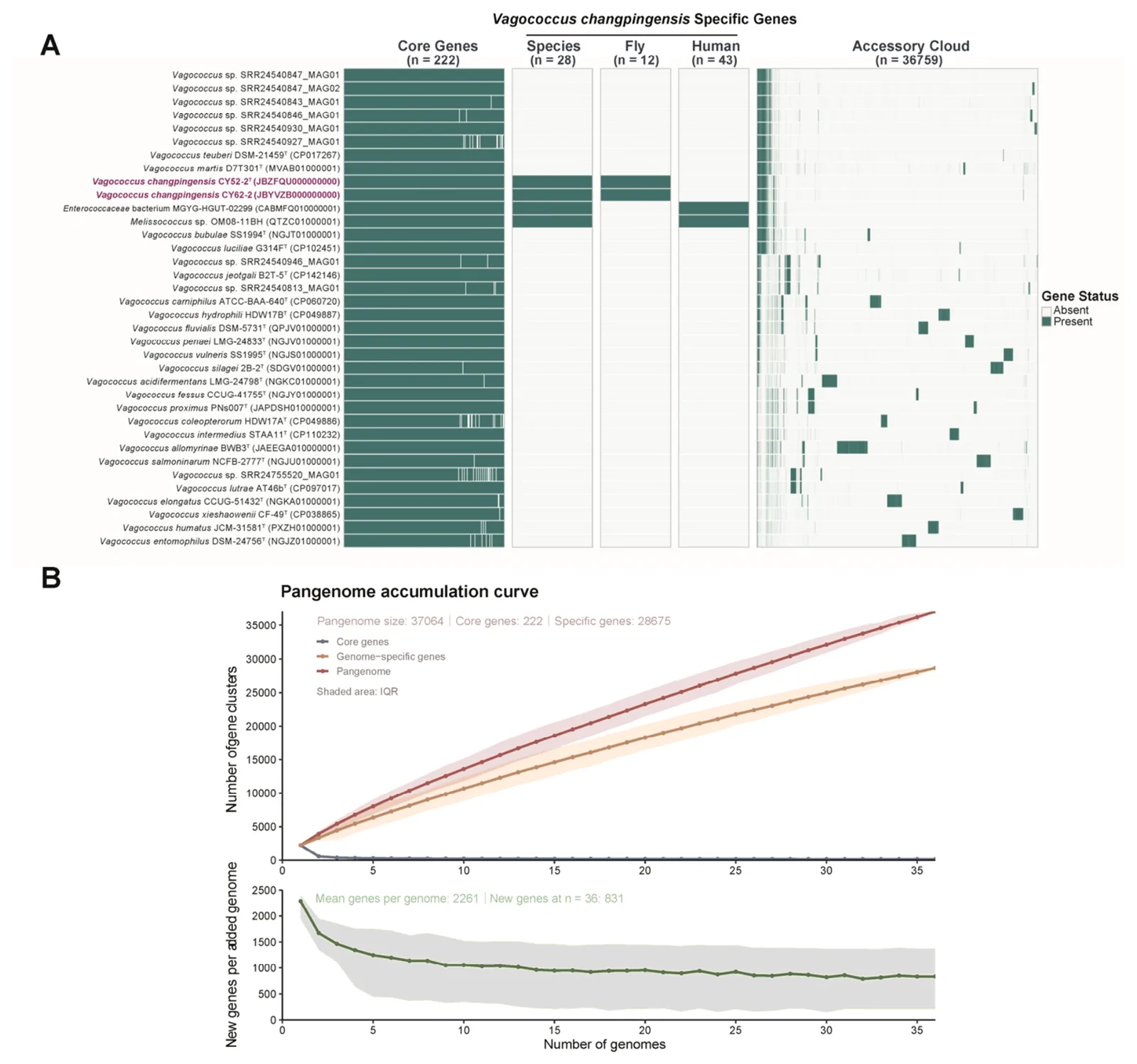
Pangenome architecture of 36 *Vagococcus* genomes. (**A**) Gene presence–absence profiles showing 222 core gene clusters, 28 *V. changpingensis*-specific gene clusters, 12 fly-associated gene clusters, 43 human-associated gene clusters, and 36,759 accessory cloud gene clusters. Teal and pale grey indicate gene presence and absence, respectively. Strains CY52-2^T^ and CY62-2 are highlighted in purple. (**B**) Pangenome accumulation analysis showing changes in the numbers of core gene clusters, cumulative genome-specific gene clusters, and total pangenome gene clusters as genomes were sequentially added. The lower plot shows the number of new gene clusters contributed by each additional genome. Shaded areas indicate interquartile ranges (IQRs). At *n* = 36, the pangenome comprised 37,064 gene clusters, including 222 core gene clusters and 28,675 cumulative genome-specific gene clusters. The mean number of genes per genome was 2,261, and 831 new gene clusters were detected upon the addition of the 36th genome. The continued increase in pangenome size is consistent with an open pangenome.

Pangenome analysis of 36 *Vagococcus* genomes, comprising the two novel isolates, the two human gut genomes, the nine fly MAGs, and the type strains of recognized species, revealed a core genome of 222 genes and an extensive accessory genome of 28,876 gene clusters, yielding a total pangenome of 37,064 gene clusters. The continuously rising accumulation curve (Figure 4) indicated an open pangenome structure, characteristic of a genus with high genomic plasticity.

Functional annotation identified clear niche-specific signatures. The 28 genes uniquely shared by all *V. changpingensis* genomes included components of arginine metabolism (e.g., argininosuccinate synthase), providing a direct genomic basis for the positive arginine hydrolysis phenotype observed in phenotypic tests. The human gut-associated genomes harboured 43 unique genes primarily linked to gastrointestinal adaptation, including L-lactate dehydrogenase and various transport systems. In contrast, the 12 genes specific to the fly-derived *Vagococcus* genomes were predominantly transposases, indicating potential for horizontal gene transfer within the fly-associated microbial community (Supplemental Table S6).

### 3.6. MALDI-TOF MS Profiling

**Figure 5:**
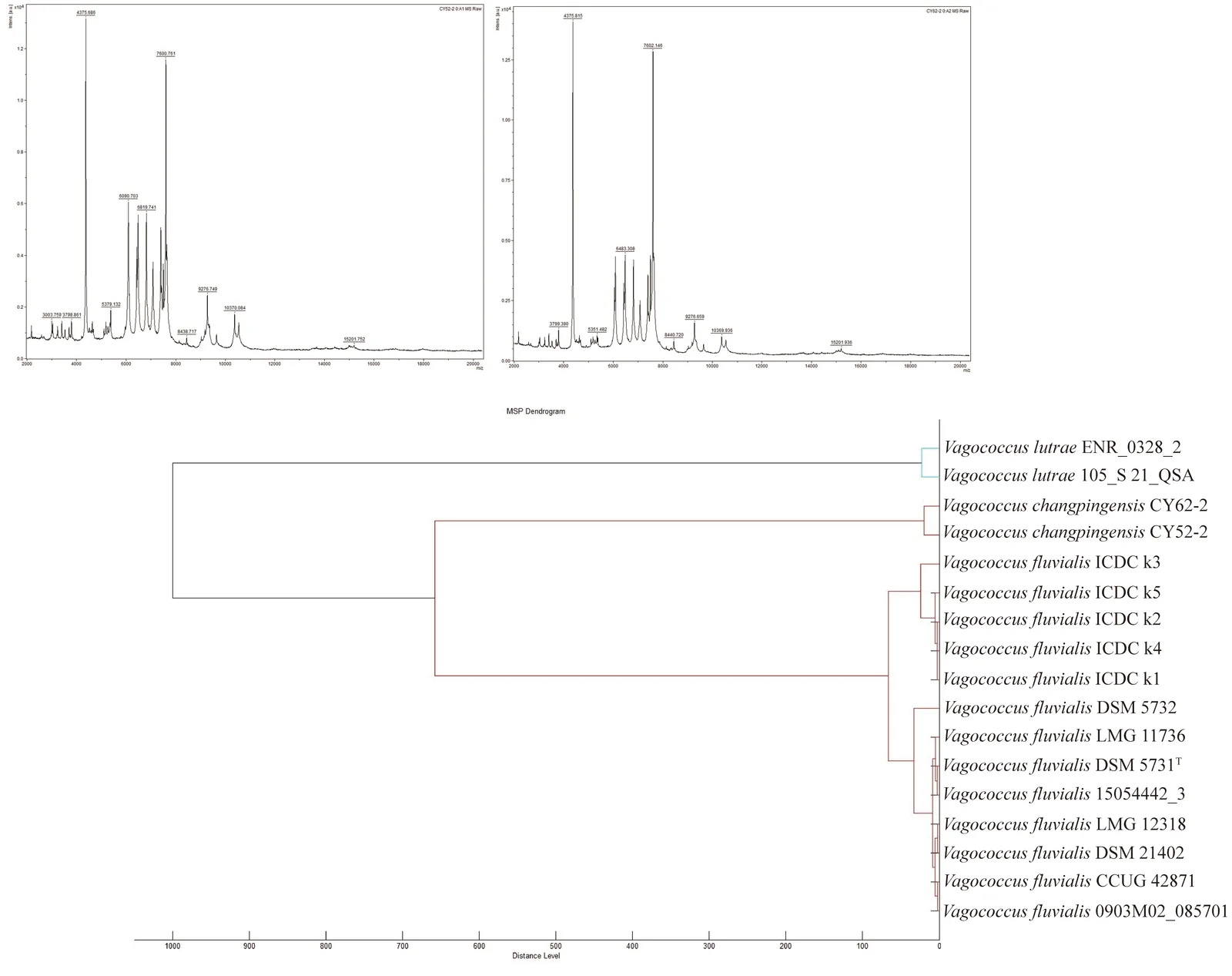
MSP dendrogram of *Vagococcus* strains based on MALDI-TOF MS spectra. MSP dendrogram generated from MALDI-TOF MS mass spectra showing the clustering relationships among strains CY52-2^T^, CY62-2, and reference strains from the Biotyper database. Strains CY52-2^T^ and CY62-2 formed a distinct cluster, separate from the two known *Vagococcus* species.

Initial MALDI-TOF MS identification using the Biotyper database failed to provide reliable species-level matches for strains CY52-2^T^ and CY62-2, attributable to the underrepresentation of *Vagococcus* species in the reference database (currently limited to *V. fluvialis* and *V. lutrae*). Main Spectrum Profile (MSP) dendrogram analysis, however, demonstrated that the two novel strains formed a single, distinct cluster clearly separated from the 15 reference strains of *V. fluvialis* and *V. lutrae* (Figure 5), further corroborating their taxonomic distinctiveness.

### 3.7. Phenotypic and Biochemical Profiles of the Novel Strains

Cells of strain CY52-2^T^ were facultatively anaerobic, Gram-positive ovoid cocci occurring in pairs, short chains, or irregular clusters (Supplemental Figure S1). Cells were non-motile. Growth occurred at 10–45 °C (optimum 35–37 °C), and weak growth was observed in the presence of 6.5% NaCl. Catalase activity was negative on blood-free media but positive on blood-containing media. Strains CY52-2^T^ and CY62-2 displayed nearly identical biochemical profiles, differing only in acid production from sorbitol (positive for CY52-2^T^, negative for CY62-2) (Table 2). Both strains were positive for the Voges–Proskauer test, leucine arylamidase, arginine hydrolysis, and acid production from ribose and trehalose, and negative for acid production from L-arabinose, mannitol, lactose, and raffinose.

Several key phenotypic features clearly differentiated the novel strains from their closest phylogenetic relatives. *V. luciliae* G314F^T^ is negative for the Voges–Proskauer test, arginine hydrolysis, and acid production from ribose and trehalose, while strains CY52-2^T^ and CY62-2 were positive for all these tests. *V. bubulae* SS1994^T^ shows positive or weakly positive reactions for acid production from L-arabinose, mannitol, lactose, and raffinose, all of which were negative in the novel strains (Table 2).

## 4. Discussion

The polyphasic taxonomic evidence presented in this study establishes strains CY52-2^T^ and CY62-2 as a novel species within the genus *Vagococcus*, for which the name *Vagococcus changpingensis* sp. nov. (chang.ping.en’sis, N.L. masc. adj., changpingensis, pertaining to Changping District, Beijing, China, where the bacterium was isolated) is proposed. Beyond the formal description of a new taxon, our findings provide a compelling example of how targeted metagenomic mining can expand the known ecological boundaries of a newly described species, extending its documented habitat from insects to the human gastrointestinal tract.

The 16S rRNA gene sequence similarities between strain CY52-2^T^ and its closest relatives, *V. luciliae* (99.6%) and *V. bubulae* (99.3%), well exceeded the conventional 98.7% species threshold, reinforcing the widely acknowledged limitation of 16S rRNA gene analysis alone for species-level resolution within the genus *Vagococcus*. In contrast, whole-genome comparisons provided unambiguous species-level discrimination. The ANI and dDDH values between the novel strains and all recognized *Vagococcus* type strains fell decisively below the 95–96% and 70% species demarcation thresholds, respectively [23]. These genomic metrics, together with the robust monophyletic clade formed in both 16S rRNA gene and core-genome phylogenies, independently validate the species novelty of *V. changpingensis*.

The identification of two human gut-derived genomes (MGYG-HGUT-02299 and OM08-11BH) as conspecific with *V. changpingensis* represents an unexpected ecological finding, extending the known distribution of this species to the human gastrointestinal tract (Supplementary Table S7). These genomes, previously deposited in public databases as *Enterococcus bacterium* and *Melissococcus* sp., respectively, highlight a broader issue: the underrepresentation of recently described or rare taxa in reference databases can lead to systematic misclassification at the genus and species levels. Our targeted metagenomic approach corrected these annotations and demonstrated that sequences attributable to *V. changpingensis* are detectable in the human gut, thereby extending the known ecological range of this species beyond its insect origin. The detection of this species in both flies and human gut samples raises the possibility of transmission through the food chain, a hypothesis consistent with the recognized role of synanthropic flies as mechanical vectors of human-associated microorganisms. However, the nature of this association, whether transient passage or persistent colonization, remains to be determined through targeted isolation from human specimens.

The absence of *V. changpingensis* from the 31 fly-associated metagenomes screened in this study, despite the recovery of diverse *Vagococcus* lineages (*V. teuberi*, *V. jeotgali*, and *V. lutrae*), suggests that this species may not be a dominant member of the fly microbiome, or that its presence is geographically or ecologically restricted. This finding highlights the value of combining culture-dependent isolation with culture-independent metagenomic exploration: the former provides the biological material necessary for formal taxonomic description and phenotypic characterization, while the latter illuminates the broader ecological context.

The pangenome analysis revealed an open pangenome structure for the genus *Vagococcus*, characterized by a small core genome and a vast accessory gene pool. This architecture underpins the ecological versatility of the genus and its capacity to colonize diverse hosts. The pangenome analysis also revealed niche-associated genomic features: arginine metabolism genes were conserved across all *V. changpingensis* genomes, gut-associated transporter genes were identified predominantly in the human-derived MAGs, and transposase genes were frequently observed in the fly-associated *Vagococcus* MAGs. These findings suggest potential functional associations with different host environments, although experimental validation is required to confirm their biological roles. The frequent occurrence of transposase genes in fly-associated *Vagococcus* MAGs is noteworthy and may reflect increased genomic plasticity in insect-associated lineages. However, whether this indicates active horizontal gene transfer or simply reflects the accumulation of mobile genetic elements in these environments remains to be determined.

The failure of MALDI-TOF MS to identify the novel strains at the species level underscores a critical practical limitation. The current Biotyper database contains spectra for only two of the 23 validly published *Vagococcus* species. We acknowledge that this comparison was limited by the reference spectra available at the time of analysis. As the clinical and ecological significance of this genus becomes increasingly recognized, the expansion of spectral databases with well-characterized type strains is essential to enable accurate and rapid identification in both diagnostic and research settings. Furthermore, the absence of *V. changpingensis* from the public fly metagenomes screened in this study does not exclude its presence in other fly populations or geographic regions, given the limited sampling available. Among the 28 genes uniquely shared by all *V. changpingensis* genomes, we identified components of arginine metabolism (e.g., argininosuccinate synthase), which provide a direct genomic basis for the positive arginine hydrolysis phenotype observed in our phenotypic assays. However, it should be noted that for most other phenotypic traits, the genetic basis has not been experimentally validated, and the observed differences remain primarily phenotypic in nature without transcriptomic or functional confirmation. Nonetheless, the arginine-associated gene-phenotype correspondence illustrates the value of integrating genomic and phenotypic data for more robust species characterization, while also highlighting the need for further functional studies to fully elucidate the genetic underpinnings of the metabolic and physiological traits of this novel species.

## 5. Conclusions

The polyphasic taxonomic data presented in this study unambiguously establish strains CY52-2^T^ and CY62-2 as a novel species, *Vagococcus changpingensis* sp. nov. Targeted metagenomic mining suggests a broader ecological distribution of this species, with genomic evidence of its presence in the human gastrointestinal tract. While the fly-to-human connection remains hypothetical, our findings highlight the utility of integrating taxonomy with metagenomics to uncover the potential ecological breadth of newly described species.

### Description of V. changpingensis *sp. nov.*

*Vagococcus changpingensis* sp. nov. (chang.ping.en’sis, N.L. masc. adj., changpingensis, pertaining to Changping District, Beijing, China, where the bacterium was isolated) is proposed. The species description is based on two strains. Based on experimental data, Cells are Gram-stain-positive, ovoid cocci that occur predominantly in pairs, short chains, or irregular clusters. Non-motile. Facultatively anaerobic. After 24 h of aerobic incubation at 37 °C with 5% CO_2_ on Columbia agar supplemented with 5% defibrinated sheep blood, colonies are approximately 1.0 mm in diameter, circular, convex, with irregular margins, greyish-white, smooth, and exhibit α-haemolysis. Growth occurs at 10–45 °C, with optimal growth at 35–37 °C. Weak growth is observed in brain heart infusion broth supplemented with 6.5% (*w*/*v*) NaCl. Catalase activity is negative on blood-free media but positive on media containing blood. Positive for Voges–Proskauer test, leucine arylamidase, and arginine hydrolysis. In API 50 CHL and API 20 Strep systems, acid is produced from ribose, trehalose, and sorbitol (type strain), but not from L-arabinose, mannitol, lactose, or raffinose. Strain CY62-2 differs from the type strain in being negative for acid production from sorbitol (Supplemental Table S8).

The type strain CY52-2^T^ has a circular chromosome of 2,019,321 bp and a plasmid of 78,932 bp, with a DNA G+C content of 33.7 mol%. The species forms a distinct phylogenetic subclade within the genus *Vagococcus* based on 16S rRNA gene and core-genome analyses. In addition to being isolated from flies, genomic sequences attributable to this species have been detected in human gut metagenomes.

The type strain, CY52-2^T^ (=GDMCC 1.17730 = JCM 33856), was isolated from a pooled fly sample collected at a retail market in Changping District, Beijing, China. The GenBank accession number for the whole-genome sequence of strain CY52-2^T^ is JBZFQU000000000.

## Figures and Tables

**Table 1:** Pairwise average nucleotide identity (ANI) values among *Vagococcus changpingensis* sp. nov., human-gut-derived database records, closely related type strains, and fly-associated MAGs.

Strain/MAG	(1)	(2)	(3)	(4)	(5)	(6)	(7)	(8)	(9)	(10)	(11)	(12)	(13)	(14)
(1) *V. changpingensis* CY52-2^T^	—													
(2) *V. changpingensis* CY62-2	99.4	—												
(3) MGYG-HGUT-02299 (SAMEA5851803)	99.4	99.3	—											
(4) OM08-11BH (SAMN09736846)	99.4	99.3	100.0	—										
(5) *V. luciliae* G314F^T^	94.1	94.0	94.0	94.0	—									
(6) *V. teuberi* DSM 21459^T^	89.5	89.5	89.6	89.6	86.0	—								
(7) *V. martis* D7T301^T^	88.6	88.4	88.5	88.5	86.3	95.6	—							
(8) *V. bubulae* SS1994^T^	89.6	89.5	89.6	89.6	90.7	84.7	84.7	—						
(9) *Vagococcus* sp. SRR24540847_MAG01	89.2	89.3	89.2	89.2	86.0	99.2	95.9	85.0	—					
(10) *Vagococcus* sp. SRR24540847_MAG02	89.2	89.3	89.3	89.3	86.0	99.2	95.9	85.0	100.0	—				
(11) *Vagococcus* sp. SRR24540843_MAG01	89.4	89.4	89.6	89.6	86.0	99.3	95.8	84.9	99.5	99.5	—			
(12) *Vagococcus* sp. SRR24540846_MAG01	89.3	89.2	89.3	89.3	86.0	99.2	95.8	84.9	99.4	99.4	99.4	—		
(13) *Vagococcus* sp. SRR24540930_MAG01	89.5	89.5	89.6	89.6	85.9	99.1	95.9	84.9	99.4	99.4	99.5	99.3	—	
(14) *Vagococcus* sp. SRR24540927_MAG01	89.2	89.3	89.4	89.4	85.9	99.3	96.0	84.9	99.5	99.5	99.5	99.4	99.4	—

Notes: Values are pairwise ANI percentages; only the lower triangular matrix is shown. All values are presented to two decimal places. ANI, average nucleotide identity; MAG, metagenome-assembled genome. BioSample identifiers are provided directly in the strain/MAG column: MGYG-HGUT-02299 is catalogued as SAMEA5851803, whereas OM08-11BH is catalogued as SAMN09736846.

**Table 2:** Phenotypic characteristics differentiating *Vagococcus changpingensis* sp. nov. from closely related *Vagococcus* species.

	1	2	3 ^a^	4 ^b^
Characteristic				
acetoin (Voges–Proskauer test)	+	+	−	−
leucine arylamidase	+	+	−	+
arginine hydrolysis	+	+	−	−
Acidification				
ribose	+	+	−	+
L-arabinose	−	−	−	w+
mannitol	−	−	−	w+
sorbitol	+	−	−	+
lactose	−	−	−	w+
trehalose	+	+	−	+
raffinose	−	−	+	+

1, *V. changpingensis* CY52-2^T^; 2, *V. changpingensis* CY62-2; 3, *V. luciliae* G314F^T^; 4, *V. bubulae* SS1994^T^. +, positive; –, negative; w+, weak positive. ^a^, Data from Guzman et al. [21]. ^b^, Data from Shewmaker et al. [22]. *V. luciliae* and *V. bubulae* are the closest phylogenetic relatives of *V. changpingensis* based on 16S rRNA gene and ANI analyses.

## Data Availability

The original data presented in the study are openly available in GenBank under the accession numbers JBZFQU000000000 and JBYVZB000000000. The type strain, CY52-2^T^ (=GDMCC 1.17730 = JCM 33856), was isolated from flies collected in a retail market in Changping District, Beijing, China.

## Supplementary Materials

The following supporting information can be downloaded at: https://www.mdpi.com/article/10.3390/microorganisms14081748/s1. Supplemental Figure S1: Cellular morphology of strain CY52-2^T^. (A) Gram staining under oil immersion showing Gram-positive ovoid cocci occurring in pairs and short chains. (B) Transmission electron micrograph (TEM) revealing detailed cell morphology and Gram-positive cell wall structure. Bar, 2 μm. Supplemental Table S1: The digital DNA-DNA hybridization (dDDH) values between the draft genomeCY52-2^T^ and its closest phylogenetic relativesby Type Genome Serve; Supplemental Table S2: The digital DNA-DNA hybridization (dDDH) values between the complete genomeof CY52-2^T^, CY62-2, and all type strains of the genus *Vagococcus*. Supplemental Table S3: CheckM-based completeness, contamination, and assembly statistics for all genomes used in this study. Supplemental Table S4: Summary of metagenomic samples, sequencing runs, and library information for all datasets from which Vagococcus metagenome-assembled genomes (MAGs) were recovered. Supplemental Table S5: Genome quality metrics of metagenome-assembled genomes (MAGs) closely related to the genus Vagococcus based on ANI analysis. Supplemental Table S6: Key specific genes identified in Vagococcus changpingensis and newly recovered metagenome-assembled genomes (MAGs) based on pangenome functional annotation. Supplemental Table S7: Available metadata for the two human gut-derived genome records included in this study. Supplemental Table S8: Biochemical characteristics of Vagococcus changpingensis CY52-2^T^ and CY62-2 with API 20strep and 50CHL.
