# Supplementary material for: Vagococcus changpingensis sp. nov., a Fly-Associated Bacterium with Human Gut Metagenomic Representatives: Genomic and Metagenomic Insights into Its Ecological Distribution
Source: Microorganisms. 2026 Aug 8;14(8):1748. doi: 10.3390/microorganisms14081748 (PMC13515212; doi:10.3390/microorganisms14081748)
Supplement: Supplementary file 1 [file microorganisms-14-01748-s001.zip › microorganisms-4455133-supplementary.pdf]

Supplemental Files:

Supplemental Figure S1: Cellular morphology of strain CY52-2^T. (A) Gram staining under oil immersion showing Gram-positive ovoid cocci occurring in pairs and short chains. (B) Transmission electron micrograph (TEM) revealing detailed cell morphology and Gram-positive cell wall structure. Bar, 2 μm.

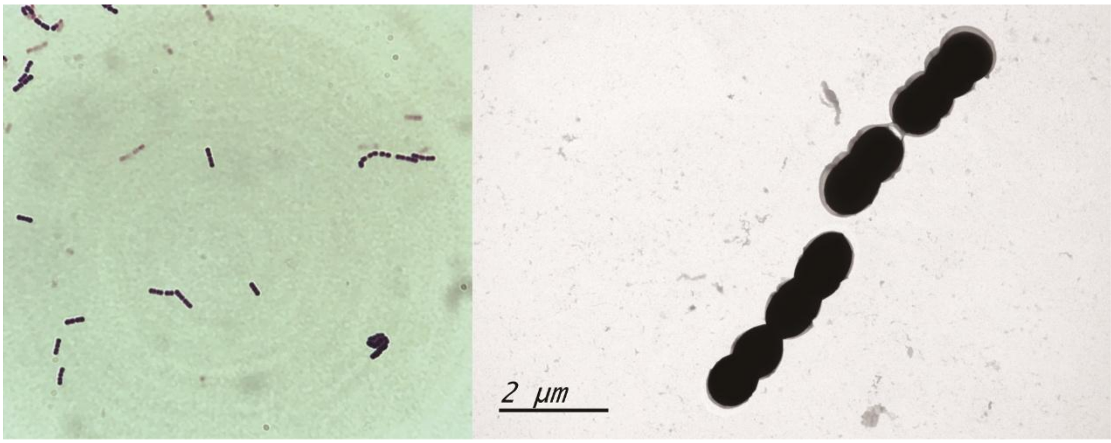

Supplemental Table S1: The digital DNA-DNA hybridization (dDDH) values between the draft genome CY52-2^T and its closest phylogenetic relatives by Type Genome Server.

Query	Subject	d4 [C.I.]	Diff. G+C %
Vagococcus changpingensis CY52-2^T	*Vagococcus changpingensi*s CY62-2	94.8 [93.1, 96.0]	0.07
Vagococcus changpingensis CY52-2^T	*Vagococcus luciliae* G314F^T	52.2 [49.5, 54.8]	0.16
Vagococcus changpingensis CY52-2^T	*Vagococcus bubulae* SS1994^T	36.6 [34.1, 39.1]	0.01
Vagococcus changpingensis CY52-2^T	*Vagococcus teuberi* DSM 21459^T	34.0 [31.6, 36.5]	0.56
Vagococcus changpingensis CY52-2^T	*Vagococcus martis* D7T301^T	31.9 [29.5, 34.4]	0.22
Vagococcus changpingensis CY52-2^T	*Enterococcus moravensi*s ATCC BAA-383^T	22.7 [20.4, 25.1]	2.43
Vagococcus changpingensis CY52-2^T	*Vagococcus acidifermentans* LMG 24798^T	22.6 [20.3, 25.1]	10.79
Vagococcus changpingensis CY52-2^T	*Enterococcus lemanii* DSM 105069^T	21.9 [19.7, 24.4]	3.68
Vagococcus changpingensis CY52-2^T	*Vagococcus penaei* CIP 109914^T	20.8 [18.6, 23.2]	1.51
Vagococcus changpingensis CY52-2^T	*Vagococcus jeotgali* B2T-5^T	20.7 [18.5, 23.2]	0.27
Vagococcus changpingensis CY52-2^T	*Vagococcus silagei* 2B-2^T	20.4 [18.2, 22.8]	1.15
Vagococcus changpingensis CY52-2^T	*Enterococcus alcedinis* CCM8433^T	20.4 [18.2, 22.8]	4.04
Vagococcus changpingensis CY52-2^T	*Vagococcus vulneris* SS1995^T	20.0 [17.8, 22.4]	1.07
Vagococcus changpingensis CY52-2^T	*Vagococcus carniphilus* SS-1714^T	19.5 [17.4, 21.9]	1.02
Vagococcus changpingensis CY52-2^T	*Vagococcus fluvialis* DSM 5731^T	19.0 [16.8, 21.4]	1.14

Supplemental Table S2: The digital DNA-DNA hybridization (dDDH) values between the complete genome of CY52-2^T, CY62-2, and all type strains of the genus *Vagococcus*.

Query genome	Reference genome (access number)	DDH*	Model C.I.
Vagococcus changpingensis CY52-2^T (SAMN55916159)	*Vagococcus changpingensis* CY62-2 (SAMN55916160)	94.9	[93.3 - 96.2%]
Vagococcus changpingensis CY52-2^T (SAMN55916159)	*Vagococcus luciliae* G314F (GCA024637875)	52.7	[50.0 - 55.3%]
Vagococcus changpingensis CY52-2^T (SAMN55916159)	*Vagococcus bubulae* SS1994 (GCA003950315)	36.8	[34.3 - 39.3%]
Vagococcus changpingensis CY52-2^T (SAMN55916159)	*Vagococcus teuberi* DSM 21459 (CP017267)	34.5	[32.1 - 37.0%]
Vagococcus changpingensis CY52-2^T (SAMN55916159)	*Vagococcus martis* D7T301 (MVAB01000001)	32.3	[29.9 - 34.8%]
Vagococcus changpingensis CY52-2^T (SAMN55916159)	*Vagococcus elongatus* CCUG 51432 (NGKA01000001)	30.6	[28.2 - 33.1%]
Vagococcus changpingensis CY52-2^T (SAMN55916159)	*Vagococcus intermedius* STAA11(GCA_029144185)	26.8	[24.4 - 29.2%]
Vagococcus changpingensis CY52-2^T (SAMN55916159)	*Vagococcus acidifermentans* LMG 24798 (NGKC01000001)	25.7	[23.4 - 28.2%]
Vagococcus changpingensis CY52-2^T (SAMN55916159)	*Vagococcus salmoninarum* NCFB 2777 (NGJU01000001)	23.4	[21.1 - 25.9%]
Vagococcus changpingensis CY52-2^T (SAMN55916159)	*Vagococcus coleopterorum* HDW17A (CP049886)	23.3	[21.0 - 25.8%]
Vagococcus changpingensis CY52-2^T (SAMN55916159)	*Vagococcus allomyrinae* BWB3-3 (GCA017829975)	23.1	[20.8 - 25.5%]
Vagococcus changpingensis CY52-2^T (SAMN55916159)	*Vagococcus humatus* JCM 31581 (GCA003950325)	22.1	[19.8 - 24.5%]
Vagococcus changpingensis CY52-2^T (SAMN55916159)	*Vagococcus penaei* CD276 (GCA001998885)	22.1	[19.8 - 24.5%]
Vagococcus changpingensis CY52-2^T (SAMN55916159)	*Vagococcus proximus* PNs007 (JAPDSH010000001)	21.8	[19.5 - 24.2%]
Vagococcus changpingensis CY52-2^T (SAMN55916159)	*Vagococcus jeotgali* B2T-5 (GCA035918315)	21.5	[19.2 - 23.9%]
Vagococcus changpingensis CY52-2^T (SAMN55916159)	*Vagococcus silagei* 2B-2 (SDGV01000001)	21.5	[19.2 - 23.9%]
Vagococcus changpingensis CY52-2^T (SAMN55916159)	*Vagococcus vulneris* SS1995 (GCA003950515)	21.2	[19.0 - 23.6%]
Vagococcus changpingensis CY52-2^T (SAMN55916159)	*Vagococcus carniphilus* ATCC BAA-640 (GCA014397115)	21.1	[18.8 - 23.5%]
Vagococcus changpingensis CY52-2^T (SAMN55916159)	*Vagococcus lutrae* CCUG 39187 (GCF003987555)	21.1	[18.9 - 23.6%]
Vagococcus changpingensis CY52-2^T (SAMN55916159)	*Vagococcus hydrophili* HDW17B (GCA011304195)	21.0	[18.8 - 23.5%]
Vagococcus changpingensis CY52-2^T (SAMN55916159)	*Vagococcus xieshaowenii* CF-210 (SRHU01000001)	20.5	[18.3 - 22.9%]
Vagococcus changpingensis CY52-2^T (SAMN55916159)	*Vagococcus fessus* CCUG 41755 (GCA_003987565)	20.2	[18.0 - 22.6%]
Vagococcus changpingensis CY52-2^T (SAMN55916159)	*Vagococcus fluvialis* bH819 (FWFD01000021)	19.5	[17.3 - 21.9%]

Vagococcus changpingensis CY52-2^T (SAMN55916159)	*Vagococcus entomophilus* DSM 24756T (NGJZ01000001)	18.6	[16.5 - 21.0%]

*Formula 2 was used.

Supplementary Table S3. CheckM-based completeness, contamination, and assembly statistics for all genomes used in this study.

Strain	Completeness (%)	Contamination (%)	Genome size (bp)	GC (%)	N50 (bp)	Contigs	Model[1]
Vagococcus changpingensis CY52-2^{T}	100	0	2,019,321	34	2,019,321	1	Neural Network
Vagococcus changpingensis CY62-2	100	0	2,025,780	34	425,686	23	Neural Network
Enterococcus bacterium MGYG-HGUT-022	100	0	2,039,736	34	473,835	20	Neural Network
Enterococcus faecalis T5^{T}	100	0.17	2,870,381	37	2,806,553	3	Neural Network
Melissococcus sp. OM08-11BH	100	0	2,039,736	34	473,835	20	Neural Network
Vagococcus sp. SRR24540813_MAG01	94.16	5.38	1,965,514	34	18,877	165	Neural Network
Vagococcus sp. SRR24540843_MAG01	99.92	3.67	2,037,946	34	28,473	117	Neural Network
Vagococcus sp. SRR24540846_MAG01	99.97	0.06	2,376,405	34	16,095	260	Neural Network
Vagococcus sp. SRR24540847_MAG01	99.47	0.02	1,915,826	34	24,844	110	Neural Network
Vagococcus sp. SRR24540847_MAG02	99.87	0.9	2,305,352	34	23,316	215	Neural Network
Vagococcus sp. SRR24540927_MAG01	94.08	0.05	1,868,292	34	21,315	130	Neural Network
Vagococcus sp. SRR24540930_MAG01	99.95	0.01	2,286,147	34	16,780	211	Neural Network
Vagococcus sp. SRR24540946_MAG01	99.02	0.4	2,199,933	34	17,118	265	Neural Network
Vagococcus sp. SRR24755520_MAG01	99.14	2.85	1,721,542	38	10,802	259	Gradient Boost
Vagococcus acidifermentans LMG-24798^{T}	100	0	2,862,533	44	155,062	40	Neural Network
Vagococcus allomyrinae WB3^{T}	99.71	4.77	5,705,793	40	252,091	43	Gradient Boost
Vagococcus bubulae SS1994^{T}	100	0.01	2,499,339	34	113,661	60	Neural Network
Vagococcus carniphilus ATCC-BAA-640^{T}	98.6	1.97	3,128,613	33	3,020,833	3	Gradient Boost
Vagococcus coleopterorum HDW17A	100	0.03	1,530,734	37	1,530,734	1	Gradient Boost
Vagococcus elongatus CCUG-51432^{T}	99.68	1.29	2,879,210	38	122,341	55	Gradient Boost
Vagococcus entomophilus DSM-24756^{T}	100	0.46	2,444,868	36	1,260,240	7	Neural Network
Vagococcus fessus CCUG-41755^{T}	98.14	0.03	2,257,832	35	823,406	8	Neural Network
Vagococcus fluvialis DSM-5731^{T}	99.99	0.5	2,651,514	32	184,338	29	Neural Network
Vagococcus humatus JCM-31581^{T}	99.12	0	2,096,134	35	408,065	10	Gradient Boost
Vagococcus hydrophili HDW17B	99.92	0.72	3,045,783	34	3,045,783	1	Neural Network
Vagococcus intermedius STAA11^{T}	99.98	0.81	2,300,692	35	2,224,209	5	Neural Network
Vagococcus jeotgali B2T-5^{T}	99.91	0.12	2,016,295	34	2,016,295	1	Neural Network
Vagococcus luciliae G314F^{T}	100	0	2,150,567	33	2,139,468	2	Neural Network
Vagococcus lutrae AT46b^{T}	99.62	0	2,081,978	38	2,058,387	3	Neural Network
Vagococcus martis DT7301^{T}	100	0	2,561,923	34	2,466,624	3	Neural Network
Vagococcus penaei LMG-24833^{T}	100	0.16	2,336,491	35	124,556	52	Neural Network
Vagococcus proximus PNs007^{T}	98.58	0.16	2,350,491	35	306,719	20	Neural Network
Vagococcus salmoninarum NCFB-2777^{T}	99.99	0.1	3,104,446	38	115,236	45	Neural Network
Vagococcus silagei 2B-2^{T}	99.96	1.25	2,525,732	35	111,189	51	Neural Network
Vagococcus teuberi DSM-21459^{T}	100	0.14	2,184,285	34	2,169,060	2	Neural Network
Vagococcus vulneris SS1995^{T}	100	0.18	2,347,966	35	121,323	43	Neural Network
Vagococcus xieshaowenii CF-49^{T}	99.98	0.24	2,071,287	36	1,983,513	3	Neural Network

^{T} indicates type strain. Completeness and contamination were assessed using CheckM (v1.2.2) with lineage-specific workflow. Models: "Neural Network" refers to the neural network

Supplementary Table S4. Summary of metagenomic samples, sequencing runs, and library information for all datasets from which Vagococcus metagenome-assembled genomes (MAGs) were recovered.

Experiment Accessio	Run ID	Sample Accessio	Organism Name	Instrument	Total Size (Mb	Library Name	Study Title (abbreviated)
SRX31741189	SRR36742111	SRS27708723	metagenome	Illumina NovaSeq X Plu	5030.77	MG-E50-336	Temperature effects on ARGs in *Musca domestica* residues
SRX31741155	SRR36742145	SRS27708689	metagenome	Illumina NovaSeq X Plu	3177.16	MG-E65-120	Temperature effects on ARGs in *Musca domestica* residues
SRX31741152	SRR36742148	SRS27708686	metagenome	Illumina NovaSeq X Plu	3417.5	MG-E65-24	Temperature effects on ARGs in *Musca domestica* residues
SRX31741145	SRR36742155	SRS27708679	metagenome	Illumina NovaSeq X Plu	3999.33	MG-E55-336	Temperature effects on ARGs in *Musca domestica* residues
SRX31143177	SRR36097650	SRS27130186	outdoor metagenome	Illumina NovaSeq 6000	1017.42	FLY_LF_DNA_2022	Flies as vectors of disease (Malaysia)
SRX31143173	SRR36097654	SRS27130182	outdoor metagenome	Illumina NovaSeq 6000	1302.84	FLY_KLCC_DNA_2022	Flies as vectors of disease (Malaysia)
ERX12872862	ERR13502892	ERS17206777	eukaryotic metagenome	Illumina HiSeq 2000	3229.12	Durcu	*Drosophila* symbiotic bacterial strains
SRX20531603	SRR24755502	SRS17840378	metagenome	Illumina HiSeq 4000	2149.46	BG2_1	Metagenome raw reads (gut)
SRX20531586	SRR24755519	SRS17840353	metagenome	Illumina HiSeq 4000	2769.58	BV4_1	Metagenome raw reads (environment)
SRX20531585	SRR24755520	SRS17840358	metagenome	Illumina HiSeq 4000	2342.42	BV3_3	Metagenome raw reads (environment)
SRX20325101	SRR24540813	SRS17648206	insect metagenome	HiSeq X Ten	1085.04	FSL_2	Dairy microbiome of *Musca domestica*
SRX20325100	SRR24540814	SRS17648204	insect metagenome	HiSeq X Ten	1233.25	FSL_1	Dairy microbiome of *Musca domestica*
SRX20325072	SRR24540842	SRS17648177	insect metagenome	HiSeq X Ten	1089.56	CVN_6	Dairy microbiome of *Musca domestica*
SRX20325071	SRR24540843	SRS17648176	insect metagenome	HiSeq X Ten	1342.12	CVN_5	Dairy microbiome of *Musca domestica*
SRX20325069	SRR24540845	SRS17648174	insect metagenome	HiSeq X Ten	594.5	CVN_4	Dairy microbiome of *Musca domestica*
SRX20325068	SRR24540846	SRS17648172	insect metagenome	HiSeq X Ten	807.44	CVN_3	Dairy microbiome of *Musca domestica*
SRX20325067	SRR24540847	SRS17648173	insect metagenome	HiSeq X Ten	840.41	CVN_2	Dairy microbiome of *Musca domestica*
SRX20324988	SRR24540926	SRS17648094	insect metagenome	HiSeq X Ten	1317.16	FSN_9	Dairy microbiome of *Musca domestica*
SRX20324987	SRR24540927	SRS17648092	insect metagenome	HiSeq X Ten	1487.68	FSN_8	Dairy microbiome of *Musca domestica*
SRX20324984	SRR24540930	SRS17648089	insect metagenome	HiSeq X Ten	975.63	FSN_5	Dairy microbiome of *Musca domestica*
SRX20324983	SRR24540931	SRS17648088	insect metagenome	HiSeq X Ten	788.31	FSN_4	Dairy microbiome of *Musca domestica*
SRX20324982	SRR24540932	SRS17648087	insect metagenome	HiSeq X Ten	1645.78	FSN_3	Dairy microbiome of *Musca domestica*
SRX20324968	SRR24540946	SRS17648073	insect metagenome	HiSeq X Ten	1246.49	CVL_1	Dairy microbiome of *Musca domestica*
SRX3099375	SRR5940682	SRS2207161	insect metagenome	Illumina HiSeq 2500	839.16	AJ141	Microbiome of blowfly *Chrysomya megacephala*
SRX3099373	SRR5940684	SRS2207159	insect metagenome	Illumina HiSeq 2500	742.7	AJ144	Microbiome of blowfly *Chrysomya megacephala*
SRX3099359	SRR5940698	SRS2207175	insect metagenome	Illumina HiSeq 2500	1203.08	AJ89	Microbiome of blowfly *Chrysomya megacephala*
SRX3099358	SRR5940699	SRS2207172	insect metagenome	Illumina HiSeq 2500	833.6	AJ135	Microbiome of blowfly *Chrysomya megacephala*
SRX3099357	SRR5940700	SRS2207174	insect metagenome	Illumina HiSeq 2500	795.81	AJ131	Microbiome of blowfly *Chrysomya megacephala*
SRX3099356	SRR5940701	SRS2207170	insect metagenome	Illumina HiSeq 2500	1046.5	AJ140	Microbiome of blowfly *Chrysomya megacephala*
SRX3099327	SRR5940730	SRS2207231	insect metagenome	Illumina HiSeq 2500	3493.01	AJ08	Microbiome of blowfly *Chrysomya megacephala* (male)
SRX3099294	SRR5940763	SRS2207133	insect metagenome	Illumina HiSeq 2500	7534.92	GL59	Microbiome of blowfly *Chrysomya megacephala* (adult)

Supplementary Table S5. Genome quality metrics of metagenome-assembled genomes (MAGs) closely related to the genus Vagococcus based on ANI analysis.

Strain	Completeness (%)	Contamination (%)	Genome size (bp)	GC (%)	N50 (bp)	Contigs	Model[1]
Vagococcus sp. SRR24540846_MAG01	99.97	0.06	2,376,405	34	16,095	260	Neural Network
Vagococcus sp. SRR24540930_MAG01	99.95	0.01	2,286,147	34	16,780	211	Neural Network
Vagococcus sp. SRR24540927_MAG02	99.94	10.71	3,210,831	34	11,359	455	Neural Network
Vagococcus sp. SRR24540843_MAG01	99.92	3.67	2,037,946	34	28,473	117	Neural Network
Vagococcus sp. SRR24540847_MAG02	99.87	0.9	2,305,352	34	23,316	215	Neural Network
Vagococcus sp. SRR24540847_MAG01	99.47	0.02	1,915,826	34	24,844	110	Neural Network
Vagococcus sp. SRR24755520_MAG01	99.14	2.85	1,721,542	38	10,802	259	Gradient Boost
Vagococcus sp. SRR24540946_MAG01	99.02	0.4	2,199,933	34	17,118	265	Neural Network
Vagococcus sp. SRR5940730_MAG01	97.57	56.89	6,085,866	35	1,561	3,876	Gradient Boost
Vagococcus sp. SRR24540813_MAG01	94.16	5.38	1,965,514	34	18,877	165	Neural Network
Vagococcus sp. SRR24540927_MAG01	94.08	0.05	1,868,292	34	21,315	130	Neural Network
Vagococcus sp. SRX31143177_MAG01	88.41	2.26	2,293,186	33	4,602	581	Gradient Boost
Vagococcus sp. SRR24540932_MAG01	84.44	2.94	1,976,141	34	15,246	184	Neural Network
Vagococcus sp. SRR24755519_MAG01	83.09	18.04	3,641,503	37	1,891	1,998	Gradient Boost
Vagococcus sp. SRR24540932_MAG02	81.58	1.25	2,130,072	34	7,352	345	Neural Network
Vagococcus sp. SRR24540932_MAG03	81.16	1.31	2,187,257	34	7,356	355	Neural Network
Vagococcus sp. SRR24540845_MAG01	80.49	8.46	1,614,327	35	3,504	514	Gradient Boost
Vagococcus sp. SRR24540931_MAG01	78.93	3.54	1,645,706	34	2,161	811	Gradient Boost
Vagococcus sp. SRR24540931_MAG02	78.23	5.8	2,608,616	36	1,990	1,377	Gradient Boost
Vagococcus sp. SRX31143173_MAG01	59.89	13.5	12,188,402	23	1,187	9,938	Gradient Boost
Vagococcus sp. SRX31741145_MAG01	53.91	1.52	924,336	39	2,611	405	Neural Network
Vagococcus sp. SRR24540842_MAG01	51.81	9.21	1,258,650	35	1,676	763	Gradient Boost
Vagococcus sp. SRR24540843_MAG02	49.07	7.48	865,549	34	1,464	585	Gradient Boost
Vagococcus sp. SRR24540814_MAG01	45.66	3.45	759,030	35	1,830	422	Gradient Boost
Vagococcus sp. SRR5940730_MAG02	43.94	10.72	1,197,834	34	3,530	338	Neural Network
Vagococcus sp. SRR24540926_MAG01	37.4	1.92	808,257	35	1,671	486	Neural Network
Vagococcus sp. SRX31741189_MAG01	34.04	1.11	781,317	39	2,370	359	Neural Network
Vagococcus sp. SRR5940698_MAG01	28.87	2.08	934,387	38	1,332	677	Neural Network
Vagococcus sp. SRX31143177_MAG02	21.2	0.16	310,623	46	1,336	227	Neural Network
Vagococcus sp. SRX31741155_MAG01	18.6	0.7	446,103	47	1,317	321	Neural Network
Vagococcus sp. SRR5940730_MAG03	16.35	6.56	2,915,374	16	1,446	2,003	Neural Network
Vagococcus sp. SRR5940684_MAG01	13.68	0.09	447,024	49	1,323	324	Neural Network
Vagococcus sp. ERX12872862_MAG02	13.09	0.07	862,041	32	1,547	546	Neural Network
Vagococcus sp. SRX31741152_MAG01	10.15	0.73	336,643	35	1,410	230	Neural Network
Vagococcus sp. SRR5940700_MAG01	9.79	2.07	2,061,060	15	1,202	1,653	Neural Network
Vagococcus sp. SRR24755502_MAG01	8.99	0.02	375,402	45	1,235	285	Neural Network
Vagococcus sp. SRR5940763_MAG01	6.65	0.64	742,716	32	1,421	516	Neural Network
Vagococcus sp. SRR5940699_MAG01	6.43	0.01	478,821	28	1,457	331	Neural Network
Vagococcus sp. SRR5940682_MAG01	6.38	0	242,588	18	1,376	175	Gradient Boost
Vagococcus sp. SRR5940682_MAG02	6.38	0	325,951	17	1,289	241	Gradient Boost
Vagococcus sp. SRR5940698_MAG02	6.38	0	269,719	20	1,553	173	Gradient Boost
Vagococcus sp. SRR5940698_MAG03	6.38	0	203,872	15	1,272	151	Gradient Boost
Vagococcus sp. SRR5940699_MAG02	6.38	0	319,503	16	1,652	196	Gradient Boost
Vagococcus sp. SRR5940700_MAG02	6.38	0	242,401	20	1,337	170	Gradient Boost
Vagococcus sp. SRR5940730_MAG04	6.38	0	255,019	20	2,044	136	Gradient Boost
Vagococcus sp. SRR5940763_MAG02	6.38	0	214,685	18	1,455	145	Gradient Boost
Vagococcus sp. SRR5940701_MAG01	6.29	0.04	1,019,003	27	1,489	689	Neural Network
Vagococcus sp. SRR5940682_MAG03	6.17	0.01	829,608	27	1,498	561	Neural Network
Vagococcus sp. SRR5940684_MAG02	5.9	0.04	1,388,517	14	1,186	1,126	Neural Network

^{T} indicates type strain. Completeness and contamination were assessed using CheckM (v1.2.2) with lineage-specific workflow. Models: "Neural Network" refers to the neural network model used in CheckM for lineage-specific bins; "Gradient Boost" refers to the gradient boosting model applied for general bins. .

Supplementary Table S6. Key specific genes identified in Vagococcus changpingensis and newly recovered metagenome-assembled genomes (MAGs) based on pangenome functional annotation.

Genomic Group	Annotated Function / Gene Description	Putative Biological Role
Vagococcus changpingensis		
(Isolates & GTDB MAGs)	Argininosuccinate synthase	Arginine metabolism / Amino acid biosynthesis
	Arginine repressor	Arginine metabolism regulation
	Tagatose-6-phosphate kinase	Carbohydrate metabolism
	HTH-type transcriptional regulator ArcR	Transcriptional regulation
	IS1182 family transposase ISEnfa2	Mobile genetic element / Genomic plasticity
	IS30 family transposase IS1062	Mobile genetic element / Genomic plasticity
	ISL3 family transposase IS1476	Mobile genetic element / Genomic plasticity
**Human gut-derived MAGs**		
(HGUT-02299, OM08-11BH)	L-lactate dehydrogenase	Anaerobic energy metabolism / Fermentation
	Anaerobic nitric oxide reductase transcription regulator NorR	Anaerobic respiration / Stress response
	PTS system mannose-specific EIID component	Carbohydrate transport and phosphorylation
	Putative phosphotransferase enzyme IIB component	Carbohydrate transport
	Putative ABC transporter ATP-binding protein YlmA	Membrane transport
	Chaperone protein DnaJ	Protein folding and cellular stress response
	Nucleotide-binding protein ExpZ	Putative transporter / Regulatory function
	IS30 family transposase IS1062	Mobile genetic element / Genomic plasticity
**Fly-derived *Vagococcus* MAGs**		
(Newly recovered SRR MAGs)	IS1182 family transposase ISEnfa2	Mobile genetic element / Genomic plasticity
	IS30 family transposase IS1062	Mobile genetic element / Genomic plasticity
	IS6 family transposase IS1216E	Mobile genetic element / Genomic plasticity

(Note: Hypothetical proteins and uncharacterized sequences have been excluded for clarity.)

Supplementary Table S7. Available metadata for the two human gut-derived genome records included in this study.

Genome record	BioSample accessio	ENA/SRA sample accessi	Sample/strain name	Organism or host	Isolation source	Geographic locatior	Collection date	Investigation/sample typ	Project
MGYG-HGUT-022[illegible]	SAMEA5851803	ERS3640329	MGYG-HGUT-022[illegible]	Enterococcaceae bacterium	human gut	China	NA	isolate	Human gut genomes
OM08-11BH	SAMN09736846	NA	OM08-11BH	Homo sapiens	feces	China: Shenzhen	2014-2-1	whole organism	NA

Supplemental Table S8: Biochemical characteristics of Vagococcus changpingensis CY52-2^T and CY62-2 with API 20strep and 50CHL

API 20strep		characteristic	*Vagococcus changpingensis* CY52-2	*V. changpingensis* CY62-2
1	VP	Acetoin (Voges-Proskaue	+	+
2	HIP	Hippurate hydrolysis	+	+
3	ESC	b-glucuronidase	+	+
4	PYRA	pyrrolidonyl arylamidase	-	-
5	α-GAL	α-galactosidase	-	-
6	β-GUR	β-glucuronidase	-	-
7	β-GAL	β-galactosidase	-	-
8	PAL	alkaline phosphatase	-	-
9	LAP	leucine arylamidase	+	+
10	ADH	Arginine hydrolysis	+	+
	Acidification			
11	RIB	ribose	+	+
12	ARA	L-arabinose	-	-
13	MAN	mannitol	-	-
14	SOR	sorbitol	+	-
15	LAC	lactose	-	-
16	TRE	trehalose	+	+
17	INU	inulin	-	-
18	RAF	raffinose	-	-
19	AMD	starch	-	-
20	GLYG	glycogen	-	-
API 50CHL	Acidification			
1	GLY	Glycerol	+	+
2	ERY	Erythritol	-	-
3	DARA	D-arabinose	-	-
4	LARA	L-arabinose	-	-
5	RIB	D-ribose	+	+
6	DXYL	D-xylose	-	-
7	LXYL	L-xylose	-	-
8	ADO	D-adonitol	-	-
9	MDX	methyl β-D-xylopyranosid	-	-
10	GAL	D-galactose	-	-
11	GLU	D-glucose	+	+
12	FRU	D-fructose	+	+
13	MNE	D-mannose	+	+
14	SBE	L-sorbose	-	-
15	RHA	L-rhamnose	-	-
16	DUL	dulcitol	-	-
17	INO	inositol	+	+
18	MAN	D-mannitol	-	-
19	SOR	D-sorbitol	+	-
20	MDM	methyl αD-mannopyranos	-	-
21	MDG	methyl αD-glucopyranosic	+	+
22	NAG	N-acetyl glucosamine	+	+
23	AMY	amygdalin	-	-
24	ARB	arbutin	+	+
25	ESC	esculin	+	+
26	SAL	salicin	+	+
27	CEL	cellobiose	-	-
28	MAL	maltose	+	+
29	LAC	lactose	-	-
30	MEL	melibiose	-	-
31	SAC	sucrose	+	+
32	TRE	trehalose	+	+
33	INU	inulin	-	-
34	MLZ	melezitose	-	-
35	RAF	raffinose	-	-
36	AMD	starch	-	-
37	GLYG	glycogen	-	-
38	XLT	xylitol	-	-
39	GEN	β-gentiobiose	+	+
40	TUR	turanose	+	+
41	LYX	D-lyxose	-	-
42	TAG	D-Tagatose	-	-
43	DFUC	D-fucose	-	-
44	LFUC	L-fucose	-	-
45	DARL	D-arabitol	-	-
46	LARL	L-arabitol	-	-
47	GNT	gluconate	-	-
48	2KG	2-ketogluconate	-	-
49	5KG	5-ketogluconate	-	-
